# Gd^3+^-DTPA-Meglumine-Anionic Linear Globular Dendrimer G1: Novel Nanosized Low Toxic Tumor Molecular MR Imaging Agent

**DOI:** 10.1155/2013/378452

**Published:** 2013-02-26

**Authors:** Tahmineh Darvish Mohamadi, Massoud Amanlou, Negar Ghalandarlaki, Bita Mehravi, Mehdi Shafiee Ardestani, Parichehr Yaghmaei

**Affiliations:** ^1^Department of Biological Science, School of Science, Science and Research Branch, Islamic Azad University, Tehran 14515-775, Iran; ^2^Department of Medicinal Chemistry, Faculty of Pharmacy and Pharmaceutical Sciences Research Center, Tehran University of Medical Sciences, Tehran 1417614411, Iran; ^3^Medical Physics and Biomedical Engineering and Nanomedicine Department, Faculty of Medicine, Shahid Beheshti Medical University, Tehran 1985717443, Iran; ^4^Department of Nanobiotechnology, Hepatitis and AIDS, Pasteur Institute of Iran, Tehran 1316943551, Iran; ^5^Department of Radiopharmacy and Medicinal Chemistry, Faculty of Pharmacy, Tehran University of Medical Sciences, Tehran 1417614411, Iran

## Abstract

Despite the great efforts in the areas of early diagnosis and treatment of cancer, this disease continues to grow and is still a global killer. Cancer treatment efficiency is relatively high in the early stages of the disease. Therefore, early diagnosis is a key factor in cancer treatment. Among the various diagnostic methods, molecular imaging is one of the fastest and safest ones. Because of its unique characteristics, magnetic resonance imaging has a special position in most researches. To increase the contrast of MR images, many pharmaceuticals have been known and used so far. Gadopentetate (with commercial name Magnevist) is the first magnetic resonance imaging contrast media that has been approved by the US Food and Drug Administration. In this study, gadopentetate was first synthesized and then attached to a tree-like polymer called dendrimer which is formed by polyethylene glycol core and surrounding citric acid groups. Stability studies of the drug were carried out to ensure proper synthesis. Then, the uptake of the drug into liver hepatocellular cell line and the drug cytotoxicity were evaluated. Finally, *in vitro* and *in vivo* MR imaging were performed with the new synthetic drug. Based on the findings of this research, connecting gadopentetate to dendrimer surface produces a stronger, safer, and more efficient contrast media. Gd(III)-diethylenetriamine pentaacetate-meglumine-dendrimer drug has the ability to enter cells and does not produce significant cytotoxicity. It also increases the relaxivity of tissue and enhances the MR images contrast. The obtained results confirm the hypothesis that the binding of gadopentetate to citric acid dendrimer produces a new, biodegradable, stable, and strong version of the old contrast media.

## 1. Introduction

Cancer is known with uncontrolled cell growth. According to the TNM staging system, cancer has 4 stages. In the early stages, cancerous cells grow in their location. These cells can gradually spread to the surrounding tissues or move to other organs. Metastasis is a stage in which cancerous cells spread to the whole body through the lymphatic system or bloodstream. It is extremely harmful and can lead to death [1]. Despite the great efforts in the areas of prevention, diagnosis, and treatment of cancer, this disease continues to grow and is still a global killer. Based on the GLOBOCAN 2008 estimates—the standard set of worldwide estimates of cancer incidence and mortality—cancer is the leading cause of death in economically developed countries and the second leading cause of death in economically developing countries [2].

Cancer has no specific symptoms. Therefore, its diagnosis can be challenging. The early diagnosis of cancer is very important in order to make the therapies more effective. In addition, a lot of these therapies are not definite. They are also costly, useless, and troublesome in metastasis stage. Thus, the key to cancer treatment is probably early diagnosis. The comparison between cancerous patients whose diseases were not diagnosed at early stages and those with early cancer detection with the same age, race, and sex shows that the survival rate increased by 85% between 1999 and 2005. Such published statistics reflect the fact that more research is needed in the area of cancer early diagnosis [3].

One of the best methods of cancer diagnosis is using molecular imaging techniques. Cancer imaging has many advantages. For example, this method does not require any tissue damage for detection and can also be a guide for biopsy. Molecular imaging includes many various techniques. CT, PET, SPECT, and MRI are considered as the most important techniques. The majority of clinical imaging systems are based on the interaction of the electromagnetic radiation with body tissues and fluids [4]. Among them, MRI (magnetic resonance imaging) is one of the most useful methods. MRI uses nuclear magnetic resonance for the visualization of the hydrogen nucleus in fat and water tissue. In cancer, damaging leads to the changes in the water content of the surrounding tumor tissues. So, MRI is widely used in medicine. In this technique, magnets make the external magnetic field so nuclear spin of the protons can be along with the magnetic field. By applying radio frequency waves and removing them, nuclei turn to the initial thermodynamical state. Signals will be received based on the different relaxation times and will convert to an image by a computer [5]. Contrast media is a material which used to enhance the image contrast. MRI contrast agents alter relaxation time of the tissue atom nucleus and thus provide a more intense signal. These materials are made based on two main metals. The most common paramagnetic metal used in contrast agents is gadolinium [6]. Gadolinium is a rare metal from intermediate lanthanide group with atomic number of 64. Free gadolinium is toxic to the human body [7]. 

Chelates are materials which have high affinity for metals. In contrast agents, chelates is used as a ligand for binding to the desired metal. The most common chelate is used to bind with gadolinium ions is called DTPA. This combination greatly reduces the toxicity risk of the free gadolinium [8].

**Figure 1:**
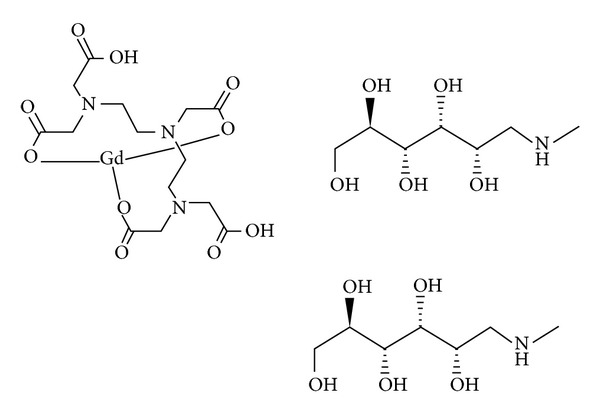
Chemical structure of dimeglumine gadopentetate.

Great efforts have been made to create a stronger, safer, and more efficient contrast agent for MRI in recent years. According to the received reports of human death due to gadolinium injection and production of poor quality and low contrast images, researchers have been persuaded to attempt to create new superior capabilities drugs. One of these contrast agents which approved by the US Food and Drug Administration is dimeglumine gadopentetate (commercial name Magnevist). This linear ionic chemical with chemical formulations of C28-H54-Gd-N5-O20 is composed of Gd^3+^, DTPA (diethylenetriamine pentaacetate), and meglumine (Figure 1). This drug is distributed in the extracellular space and does not cross the blood-brain barrier. This compound is the oldest contrast agent in the market world and is still highly functional in MRI [8].

Nanomedicine is the application of nanotechnology in medicine. Among the wide applications of nanomedicine, one of them is the use of nanoparticles in *in vivo* imaging. Since nanosized particles are compatible with biological systems and their large surface area to volume ratio makes them capable of drug loading and conjugation, nanoparticle contrast media in MR imaging have been used to enhance the properties of macroscopic counterparts. So far, they have been enabled to create images with desirable contrast in several cases [9]. 

Dendrimers are repetitively branched nanopolymers. These materials are usually symmetrical around a central core and have often three-dimensional spherical morphology. Using dendrimer has recently become an increasingly important part of MRI contrast agent in pharmaceutical studies. The properties of dendrimers are distinguished by the functional groups on the surface; however, there are examples of dendrimers with internal functionality which means that the dendrimers incorporate the functional groups in their inner space. Unlike most polymers, dendrimers can be water-soluble. Dendrimers terminated in hydrophilic groups are soluble in polar solvents. Other controllable properties of dendrimers include toxicity, crystallinity, and chirality. They also have some unique features due to their globular shape and the presence of internal cavities [10, 11].

One of the most important difficulties in the usage of dendrimer as a contrast media is its high toxicity in the body. To lessen this problem, many strategies have been proposed and applied so far. The two main strategies are designing and synthesis of biodegradable dendrimers and surface engineering. The first strategy is performed using biodegradable cores or surface units which are intermediates of the metabolic pathways, and the second one is carried out by PEGylation, acetylation, and so forth [12]. In synthesis of dendrimer used in this research (Namazi and Adeli method [13]), both strategies were applied. PEG is used as the core of dendrimer and its surrounding groups are citric acids (the citric acid cycle intermediates). As a result, the dendrimer is probably biodegradable and has no significant toxicity. 

In this research, for the first time dimeglumine gadopentetate was loaded and/or conjugated to the anionic linear globular nanosized dendrimer (ALGND) in order to create a novel MRI contrast agent with greater stability, fewer side effects (toxicity), and enhanced tumor image contrast compared to standard drug Magnevist.

## 2. Methods

### 2.1. Material

Gadolinium(III) chloride (99.99%),* N*-hydroxysulfosuccinimide sodium salt (≥98%), Diethylenetriamine pentaacetic acid (≥97%), and amino salicylic acid (99%) were purchased from Aldrich Co. Phosphate-buffered saline (powder, pH 7.4), adipic acid dihydrazide (≥98%), Meglumine diatrizoate, diclofenac sodium salt, and mefenamic acid were obtained from Sigma Co. 1-Ethyl-3-(3′-dimethylaminopropyl) carbodiimide, citric acid (≥99%), pyridine (≥98%), and thionyl chloride were obtained from Merck Co.

### 2.2. ALGND Preparation

The dendrimer was of the first generations of anionic linear globular copolymer type. Using the method introduced by Namazi and Adeli, this dendrimer was synthesized in two steps including esterification and combination with citric acid. The monomer units of ester-liked fragment were citric acid and diacyl halide poly(ethylene glycol) was used as the dendrimer core. The final complex is CA-PEG-CA triblock copolymer. Though most of synthetic triblock polymers are nonbiodegradable and have toxic effects on the body, the synthesized dendrimers produced using citric acid and poly(ethylene glycol) have none of these problems and are suitable to use in contrast media combination [13].

### 2.3. Synthesis of Gadopentetate Dimeglumine (Gd^3+^-DTPA-meglumine)

Gadopentetate was synthesized using 15 mL water as a solvent, DTPA as the ligand, and GdCl_3_. Hot-Plate and magnetic-stirrer machine was used to complete the reaction. Finally, in order to increase the stability of the solution, meglumine combination as a coligand was added. All the amounts were measured using an analytical scale with an accuracy of 0.0001 g. The reaction between DTPA, GdCl_3_, and meglumine produced gadopentetate dimeglumine (Gd^3+^-DTPA-meglumine complex). 

### 2.4. Creating Gd^3+^-DTPA-meglumine-dendrimer

Following the adding of 5 mg EDC powder, 1 mL of ALGND with a concentration of 20 g/L was inserted to vial of 15 mL gadopentetate dimeglumine (as mentioned before, this powder was purchased from Sigma Company). EDC is a carboxyl group activator and it was used to activate carboxyl end of the complex (Gd^3+^-DTPA-meglumine). After that, 5 mg of ADH (adipic acid dihydrazide) and N-Sulf-HS (N-hydroxysulfo-succinimide) were added to the solution. Mechanical severe shake (sonicator machine) was used to increase the level of contact surface. Using the ultrasound waves, the particles in the solution were agitated. This method is usually used in nanotechnology in order to disperse the nanoparticles in the solution, evenly. In order to purify the obtained product, dialysis bag with a cut-off point of 1000 Da and water as the solvent were use. Finally, these two compounds (standard and nanosized Magnevist) were changed into powder for greater stability by freeze-dryer machine. (The standard Magnevist had a gelatinous state.)

### 2.5. Quality Control

Quality control tests of this research include measuring the basic characteristics of the drug such as its gadolinium content, chemical structure, and molecular weight. The gadolinium content of gadopentetate was measured by ICP-AES machine. The spectroscopy studies included liquid chromatography mass spectrometry (LC-MS) and Fourier transform infrared spectroscopy. For each, lyophilized standard Magnevist was applied.

To determine and compare the elemental composition of the standard and nanosized Magnevist, CHN analyzer SERIES II model manufactured by Perkin Elmer Co., was used.

### 2.6. Measurement of the Size and Zeta Potential Distribution

Determination of the zeta potential and size of the drugs was performed using Zeta sizer Nano ZS model manufactured by Malvern in England which had the measurement range from 6 nm to 0.6 *μ*m, zeta potential measurement range from 120 mV to +120 mV, and molecular weight measurement range from 10 to 2 × 10^7^ Daltons.

### 2.7. Microscopic Study

Microscope imaging of this research includes AFM (atomic force microscope). In this study, the images were developed of the sample drug of nanosized Magnevist.

### 2.8. Cellular Studies

For the cellular studies, HEPG2 cells (human liver hepatocarcinoma cell line) were used. DMEM was the primary culture medium. The culture medium contained 5 mL FBS 10%, 0.5 mL penicillin and streptomycin mixture, 5 mL glutamine amino acid, and 0.5 mL antibiotics. After the proper cells' growth, they were separated through trypsin enzymes and FBS solution and were centrifuged at 2000 rpm for 5 minutes.

### 2.9. Cell Uptake

Having the cells being counted, the required volume for the study was calculated (200 thousand cells). 6-well plates were used to determine the entry of the drugs into cells. 50 *λ* cells (obtained through calculating) and 4 cc culture medium were added to the wells, 200 *λ* of gadopentetate dimeglumine (Magnevist) was injected to 3 wells while to the 3 other wells 200 *λ* gadopentetate dimeglumine-dendrimer (nanosized Magnevist) was injected (repeated 3 times). Another plate was used for the control cell and nothing was injected into it. Pipetting process was carried out to mix the cells with the drug. The plates were put in 37°C incubator for 90 minutes until the drug was properly exposed to cells. The content of each well was transferred to a tube and centrifuged at 1500 rpm from 10 to 15 minutes. As much as 2 mL of culture medium was added to the sediments and cells were decomposed using acid. Afterwards, the gadolinium concentration of the tubes was measured through ICP-AES technique. The control solution was used for machine calibration. In this method, gadolinium was considered as a detector. The more gadolinium detected in cell, the more drugs entered it.

### 2.10. MTT Assay

The procedures described above were repeated for the cell separation and counting. This time, in order to determine cytotoxicity of the drugs, 96-well plate was used. 20 *λ* cells (the calculated amount) and 80 *λ* culture medium were added to 21 wells. 3 doses of standard Magnevist and nanosized Magnevist (25, 50, and 100 *μ*L) were injected into 18 wells (repeated for three times) and no drug was added to the other 3 (control group). The total volume reached 200 *μ*L by adding the additional culture medium. The plate was put in 37°C incubator for 24 hours. After 24 hours, 20 *μ*L of MTT solution was added to each well. The plate was covered with an aluminum foil (reaction in darkness) and was put in 37°C incubator for 3 hours. Then, the supernatant was discarded and 100 *μ*L of DMSO and NaCL solution was added to each well. In order to dissolve MTT in the whole solution, the plate was placed on a shaker for a few minutes. Finally, their absorption was read by ELAISA reader machine at 570 nm. More absorption shows more cell viability.

### 2.11. Magnetic Resonance Imaging

Magnetic resonance imaging was performed using 3 T MRI machine in *in vitro* and *in vivo *environments.

### 2.12. *In Vitro* MRI Study

**Figure 2:**
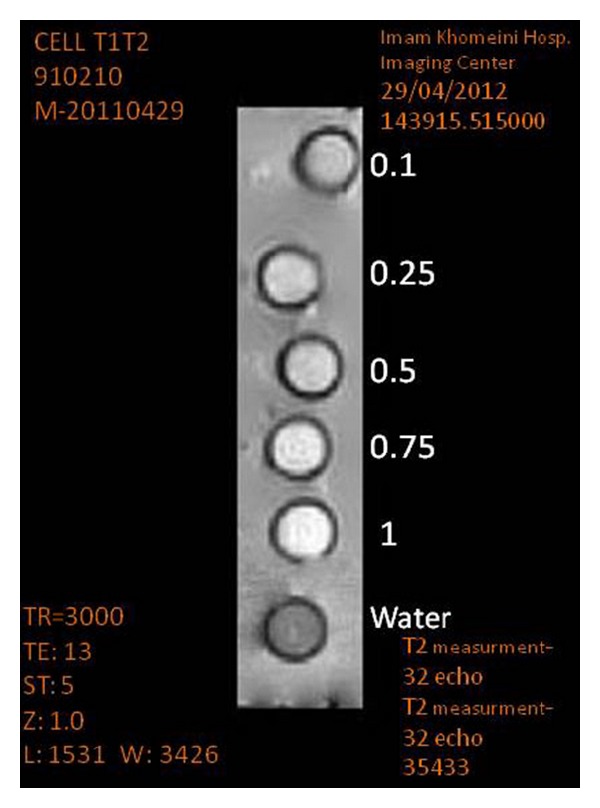
Schematic illustration of performance of *in vitro T*_1_/*T*_2_ measurements in 5 doses.

5 doses of synthesized nanosized Magnevist (0.1, 0.25, 0.5, 0.75, and 1 molar) were prepared (Figure 2). Water was used as a control group. 3 T MR imaging was conducted using the following protocol:

 Standard Spin Echo, # of Echoes  = 32,
 TE = 13/26/39/52/66/79/92/105/
 118/145/158/171/184/198/211/237/250/264/
 277/290/303/330/343/356/369/382/396/422 ms,
 TR  =  20/50/100/200/400/2000/3000 ms,
 Matrix = 256 ∗ 256,
 Slice Thickness = 1/5 mm,
 FOV = 18 ∗ 18 cm,
 NEX = Non.

There are two main mechanisms in magnetic resonance imaging: Spin-lattice relaxation and spin-spin relaxation. Times characterizing these two mechanisms are called spin-lattice relaxation time (*T*_1_) and spin-spin relaxation time (*T*_2_). The former time indicates the thermodynamic equilibrium of magnetization, but the latter time shows exponentially signal decay. The signals of the images were obtained using Dicomworks software (1.3.0.5 version) in 28 TE (echo delay time) and 7 TR (repetition time). Finally, computing the relaxation times of *T*_1_ and *T*_2_ was done using MATLAB (1.0.0.1 version) and Microsoft Office Excel 2007 software.

### 2.13. *In Vivo* MRI Study (Animal Model)

One adult (8 weeks old) male breast cancer model mouse was used for the study. Initially, 0.1 mL of anesthetic drug (mixture of ketamine and xylazine) was given to the mouse. Then a whole body MR imaging (at 3 T) was conducted. Afterwards, 0.2 mL of nanosized Magnevist was injected into the mouse (IV injection). A whole body MR imaging (at 3 T) was conducted again to compare the contrast of the image with the previous one. The images were visualized using MicroDicom (0.7.1.1824 version) software.

## 3. Result and Discussion

### 3.1. Synthesis

**Figure 3:**
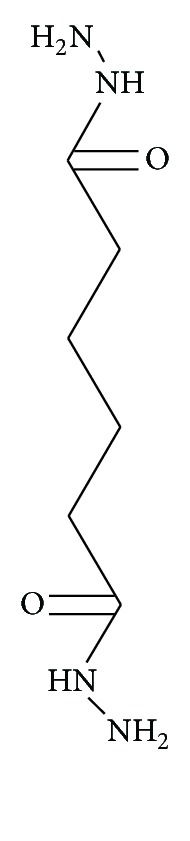
Chemical structure of ADH.

**Figure 4:**
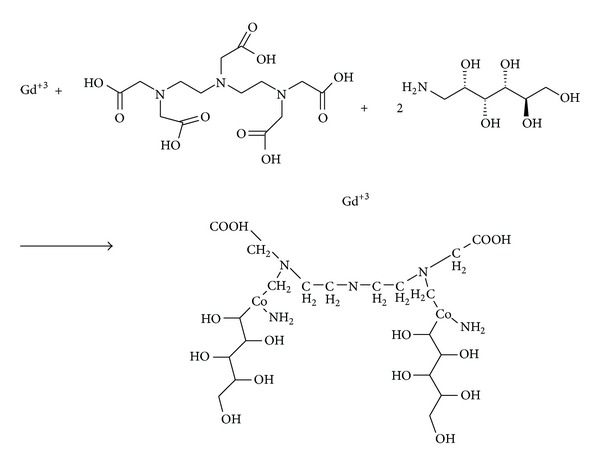
The reaction between Gd^3+^, DTPA, and meglumine to form gadopentetate dimeglumine.

**Figure 5:**
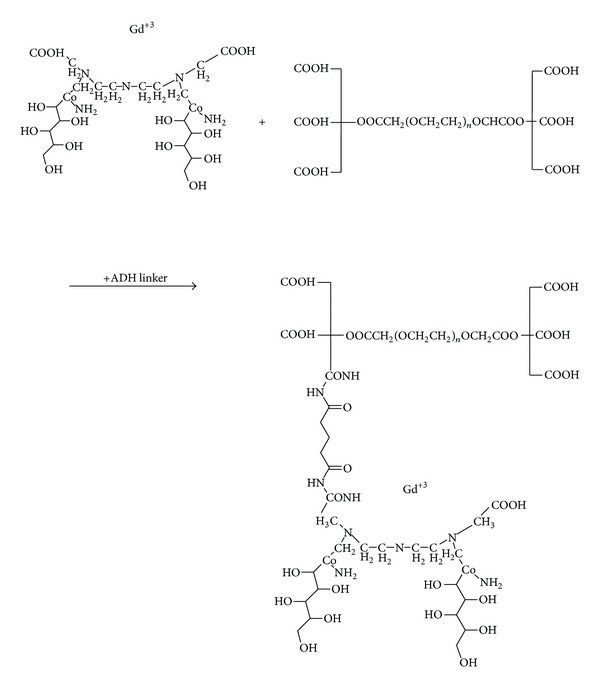
The final reaction—connecting the drug to dendrimer.

Figures 4 and 5 show the chemical reactions between Gd^3+^, DTPA (diethylene triamine pentaacetic acid), 2 meglumine (N-methyl glucamine), ADH (adipic acid dihydrazide), and ALGND. Firstly, the complex of Gd and DTPA is formed. Then, the coligand (Meglumine) is conjugated with the complex for greater stability. The result is an amide compound which is known as gadopentetate dimeglumine (Gd-DTPA-meglumine). After that, the ALGND is loaded and/or conjugated with the compound by the help of EDC (a carboxyl group activator), N-sulf-HS (removal of water effect), and ADH (the liker). EDC activates the carboxyl groups for the amidation reaction.  Figure 3 shows the chemical structure of the ADH. It has 2 amine groups at the two ends. One of them is conjugated with free carboxyl group of DTPA and the other one is conjugated with carboxyl group of citric acid in ALGND. Besides the conjugation, gadopentetate dimeglumine can be trapped into dendrimer pores (loading). Therefore, the final compound will be the new drug (Gd^3+^-DTPA-meglumine-dendrimer).

### 3.2. Quality Control Tests

#### 3.2.1. Gadolinium Content

The gadolinium content of the gadopentetate is shown as mean ± SD in Table 1. The number is almost the same number as it has been reported [14].

#### 3.2.2. Spectroscopy Studies

**Figure 6:**
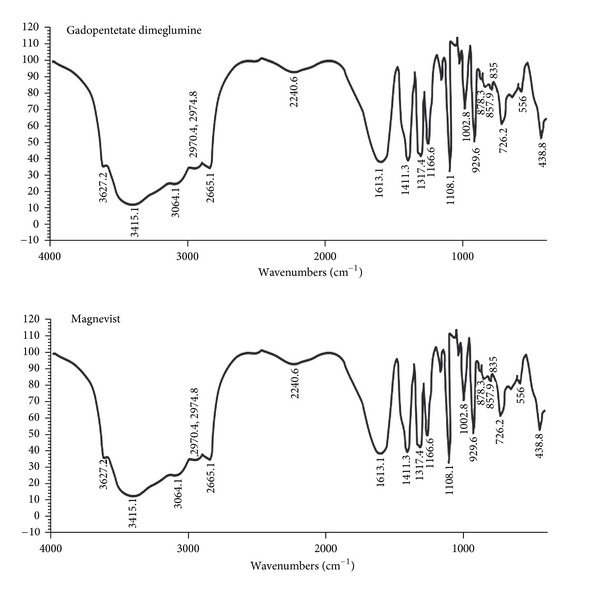
FT-IR spectrum of the synthetic and standard Magnevist.

**Figure 7:**
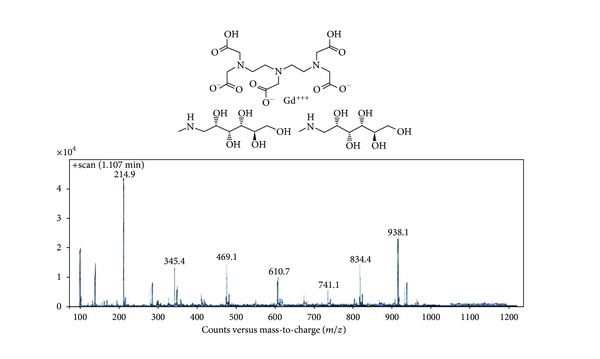
The LC-MS spectrum of Magnevist.

Figures 6 and 7 indicate the FT-IR and LC-MS spectrum of standard Magnevist. As it can be seen, the FT-IR spectrum of the synthetic Magnevist, and standard one is compared. The accordance confirms the proper synthesis. The wide peak in 3000–4000 cm^−1^ shows the presence of hydroxyl and carboxyl groups. The observed peak in 2700–2900 cm^−1^ is related to the type and nature of aliphatic hydrogen and carbon of the compound. Peaks existing in 1500–1600 cm^−1^ are related to NH groups. The number reported in LC-MS spectrum (938.10000) shows the molecular weight of the compound and it is the exact number of the reported molecular weight [8].

#### 3.2.3. CHN Analysis

Determining the C%, H%, and N% of the standard and nanosized gadopentetate by CHN analyzer confirms the proper synthesis. As it can be observed in Tables 2 and 3, the numbers calculated from CHN analysis data are in accordance with molecular formula.

#### 3.2.4. Measurement of the Size and Zeta Potential of the Compounds

**Figure 8:**
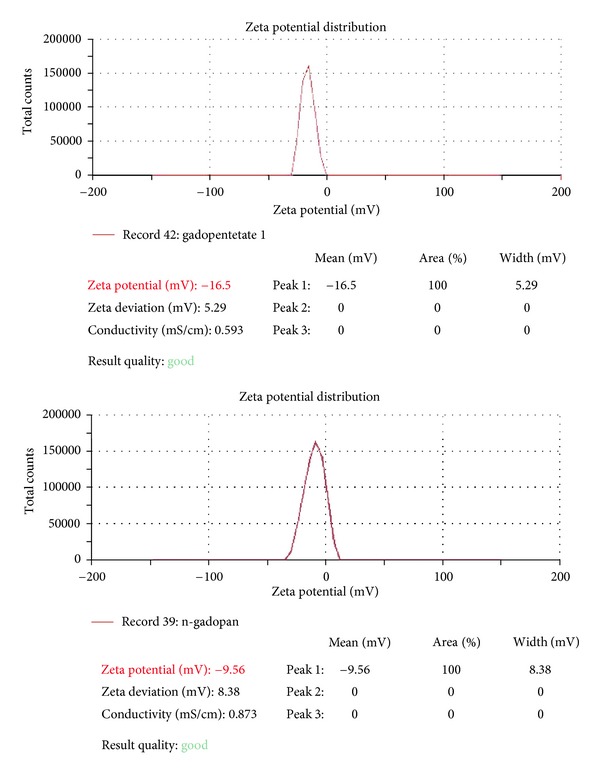
The zeta potential of gadopentetate and nanosized gadopentetate (mV).

The zeta potential of Magnevist and nanosized Magnevist are compared with each other. As it was expected, attaching the drug to dendrimer reduces the charge of final compound (−16.5 to −9.56 mV). The ALGND has negative charge on its surface, so it can reduce the total drug charge and, as a result, the drug can penetrate a cell (Figure 8).

**Figure 9:**
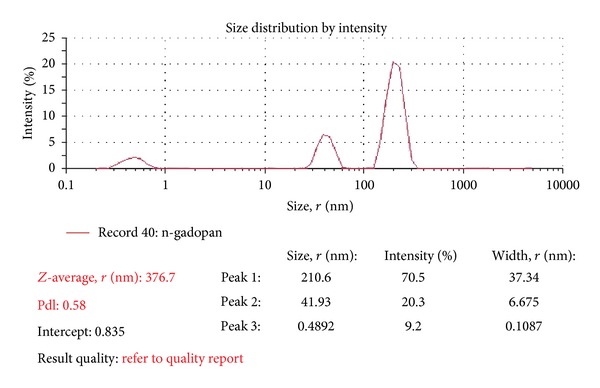
Size of the nanosized drug.

The size of the nanodrug is 41.93 nm according to the size measurement of zeta sizer (Figure 9).

#### 3.2.5. Microscopic Study

**Figure 10:**
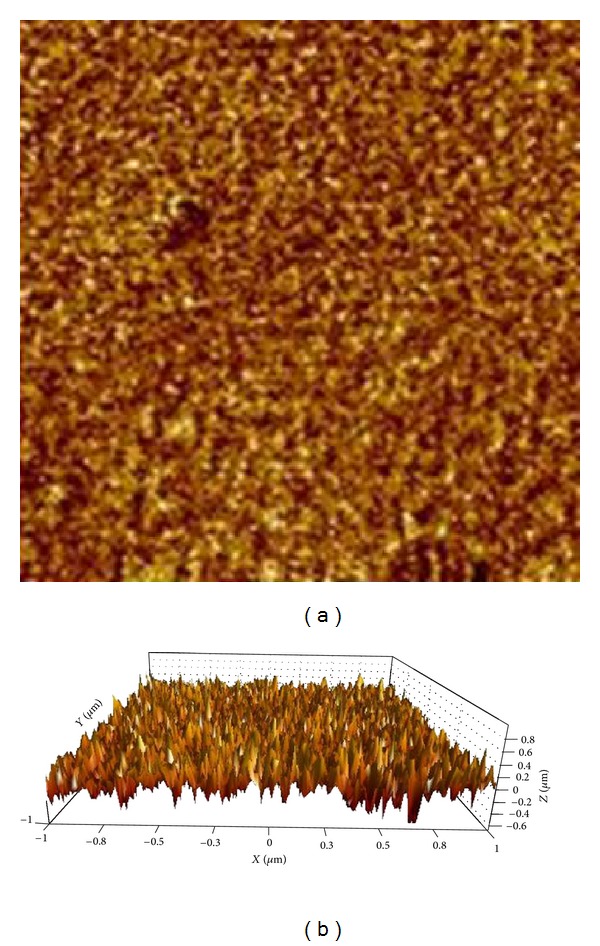
AFM images. (a) Two-dimensional AFM image of nanosized gadopentetate. (b) The three-dimensional image.

AFM imaging of the nanosized drug is shown in Figure 10. The calculated size in AFM is 22 nm. The size differences obtained from zeta sizer and AFM can be because of the different conditions of the sample in these 2 methods. In AFM, the sample is dry; however, in zeta sizer, it is a solution, so aggregation is a possibility for this result.

#### 3.2.6. Cell Uptake

**Figure 11:**
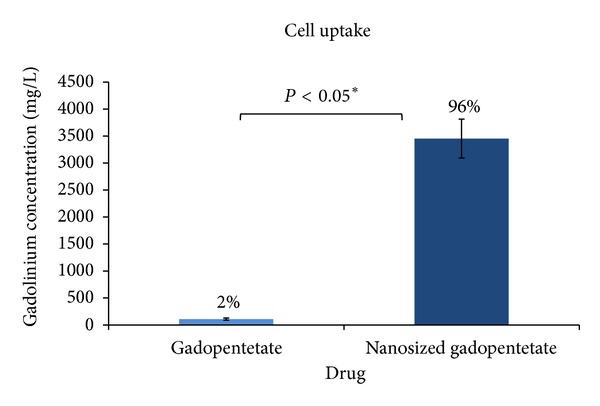
The cell uptake of the standard drug in comparison with nanosized one.

The standard Magnevist is an extracellular contrast media, so it is not able to enter a cell [15]. Attaching the drug to dendrimer makes it an intracellular contrast media. As it can be observed in Table 4 and Figure 11, significant amount of the drug could enter the HEPG2 cell line. The synthetic drug is able to penetrate into the cell probably due to its nanoscale size and total charge reduction (Figure 8). The mechanism of this entry is receptor-mediated endocytosis. Therefore, ALGND carries the drug into the cell.

#### 3.2.7. MTT Assay

**Figure 12:**
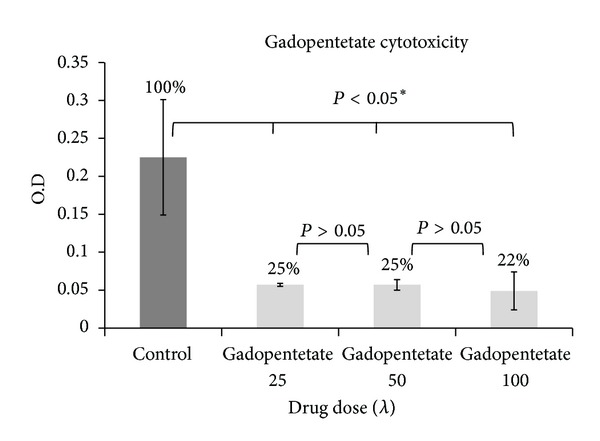
The cytotoxicity of standard gadopentetate (the cell viability is written on the top of each graph).

**Figure 13:**
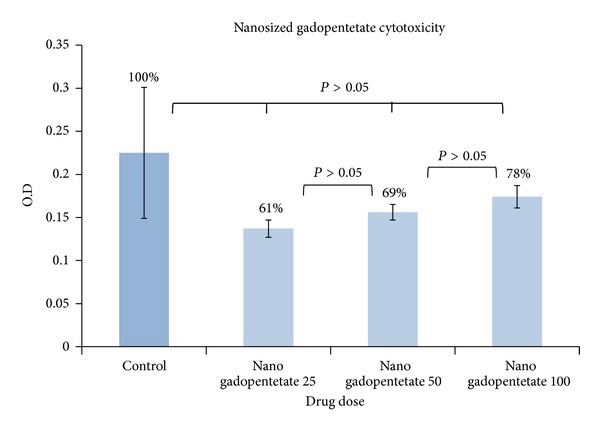
The cytotoxicity of nanosized Gadopentetate (the cell viability is written on the top of each graph).

**Figure 14:**
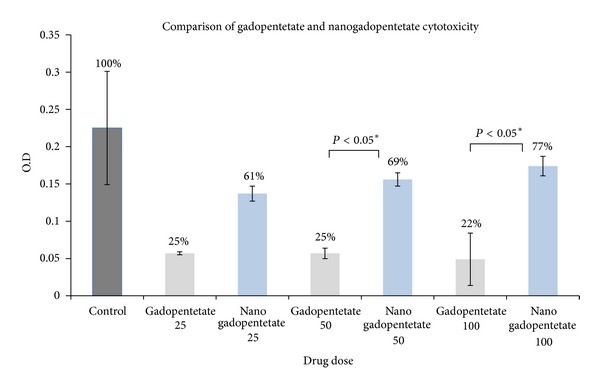
The final comparison between the cytotoxicity of standard and nanosized drugs.

Table 5 and Figure 12 show the cytotoxicity of gadopentetate and Table 6 and Figure 13 show the cytotoxicity of nanosized gadopentetate.  Table 7 and Figure 14 indicate a total comparison between the results. As it can be seen in Figure 14, cytotoxicity (in HEPG2 cell line) is reduced in nanosized drug compared to standard drug in 50 and 100 doses significantly (*P* < 0.05). surprisingly, it was observed that the cell viability is enhanced by increasing the drug dose insignificantly (*P* > 0.05).

### 3.3. *In Vitro* MRI Study

#### 3.3.1. *T*_1_ Measurement

**Figure 15:**
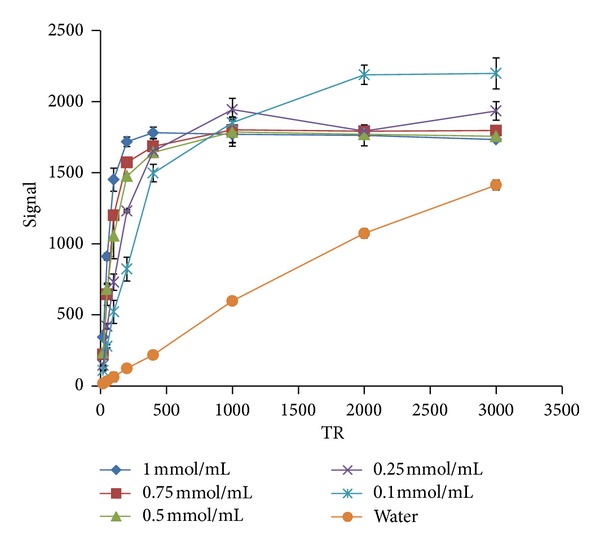
Signal relative to TR—the *T*_1_ graph.

**Figure 16:**
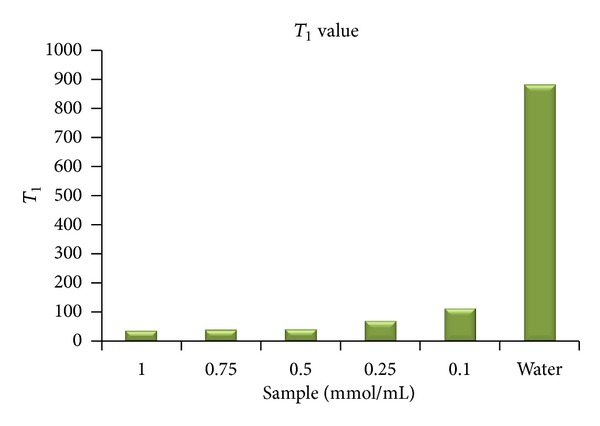
*T*_1_ value relative to the drug concentration.

The *T*_1_ graph (signal relative to TR) is shown in Figure 15. The lines show the different doses compared to water. In Table 8, *T*_1_ is calculated via (1) and using MATLAB software.  Figure 16 also shows the impact of the synthetic drug on reduction of the *T*_1_ value in comparison with water. As it can be observed, the drug can reduce *T*_1_ by increasing its doses significantly:

(1) $$\begin{array}{c} \text{Signal}=S_{0}\left(1-e^{-\text{TR}/T_{1}}\right). \end{array}$$

#### 3.3.2. *T*_2_ Measurement

**Figure 17:**
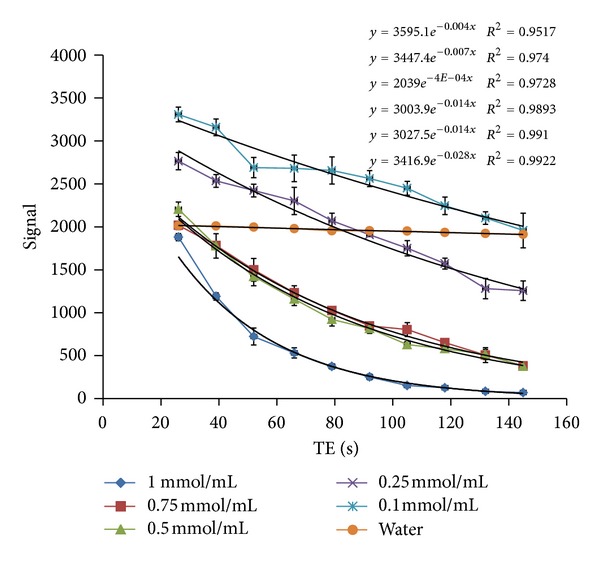
Signal relative to TE in 5 doses of the drug compared to water. The equation and *R*-squared value are shown on the right top of the graph, respectively.

**Figure 18:**
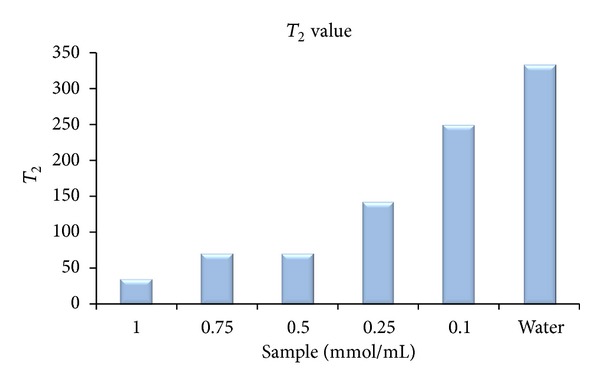
*T*_2_ value relative to the drug concentration.

The *T*_2_ graph (signal relative to TE) is shown in Figure 17. The lines show the different doses compared to water. In Table 9, *T*_2_ is calculated via (2) and Microsoft Excel software. Figure 17 also shows the impact of the synthetic drug on reduction of the *T*_2_ value in comparison with water. As it can be observed, the drug can reduce *T*_2_ by increasing its doses significantly (Figure 18). It is clear that the drug reduces the *T*_1_ value more than *T*_2_ value. So, probably it is a *T*_1_ MRI contrast agent type:

(2) $$\begin{array}{c} \text{Signal}=S_{0}e^{{-T}_{2}\cdot \text{TE}}. \end{array}$$

#### 3.3.3. Relaxivity

**Figure 19:**
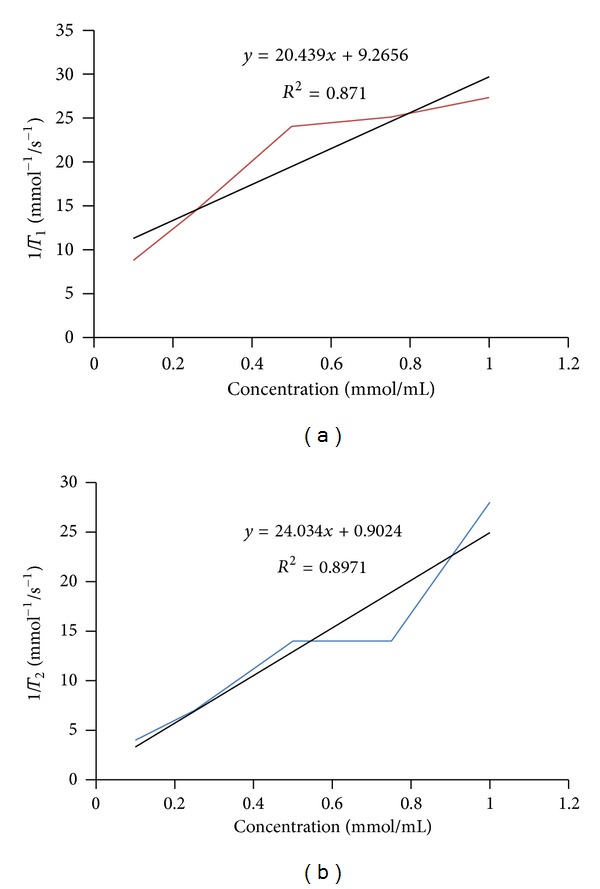
The relaxivity—(a) *R*_1_ and (b) *R*_2_.

Relaxivity is calculated via the graph in Figure 19. As it can be noticed, the relaxivity is enhanced by increasing the drug dose (linear relation). Obtaining the linear equation from Microsoft Excel, the slope of the line is the total relaxivity of the drug. *R*_1_ is 20.44 mM^−1^s^−1^ and *R*_2_ is 24.03 mM^−1^s^−1^. The *R*_1_ and *R*_2_ of Magnevist were reported to be 1.5 and 2.9 mM^−1^s^−1^, respectively [15], so the synthetic drug can increase the relaxivity significantly.

### 3.4. *In Vivo* MRI Study

**Figure 20:**
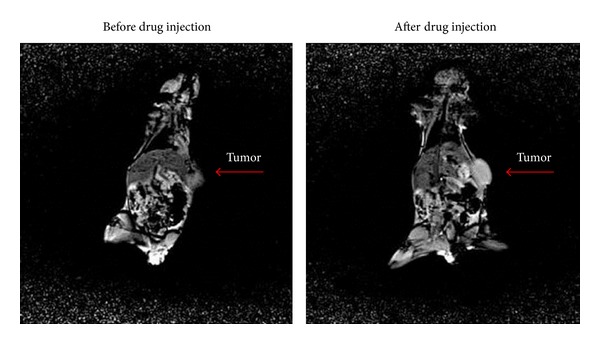
The *in vivo* study—the tumor is more recognizable after drug injection. The same image as before injection was obtained for Magnevist injection.

The result of the *in vivo* imaging is shown in Figure 20. The tumor is marked. As it can be seen clearly, the image contrast is enhanced and the tumor is more recognizable after drug injection.

## 4. Conclusion

The unique characteristics of the anionic linear globular dendrimer-G1 loaded and/or conjugated on Magnevist make it a greater contrast media. The new drug is more stable, more soluble, and biodegradable, has the ability to enter the HEPG2 cell line, and has low cytotoxicity. 

Generally, one of the main problems with the conjugation of the drug to dendrimer surface is insolubility of the final solution [16]. The dendrimer used in this research can take a large amount of a drug and be still soluble because of PEG in its core. In addition, PEG has anticancer effect and tendency to cancerous tissues. So, without any targeting agent, even though weakly, PEG can typically play the role of the targeting agent. The surrounding citric acid branches also give it negative charge. So, it does not interact with cell surface and does not destroy the cell membrane. Besides, citric acid is one of the intermediates of metabolic pathways and is metabolized in body so quickly that it does not have toxic effects in the body and makes the entire drug biodegradable.

Even though the results obtained from this research are very promising, further studies are required. If a targeting agent such as monoclonal antibodies, peptides, and aptamers is loaded on the dendrimer surface, more specific contrast media will be created. 

## Figures and Tables

**Table 1:** The comparison of gadolinium content in our synthetic gadopentetate and the reference.

Gadolinium (mg)	Gadopentetate (mean ± SD)	Reference [14]
	76.55 ± 4	78.63

**Table 2:** CHN analysis result of gadopentetate.

Element	C	H	N
Percent of element (%)	33.02%	6.41%	6.92%
Number of element	26	60	5
Chemical formula: C_28_H_54_GdN_5_O_20_			

**Table 3:** CHN analysis result of nanosized gadopentetate.

Element	C	H	N
Percent of element (%)	33.14%	6%	6.95%
Number of element	58	125	10
Chemical formula: C_61_H_102_GdN_9_O_41_			

**Table 4:** The cell gadolinium content of the gadopentetate and nanosized gadopentetate.

Drug	Gd concentration (mean ± SD)
Gadopentetate	108.82 ± 20
Nanosized gadopentetate	3452.77 ± 360

**Table 5:** The O.D reported by ELASA Reader in 3 gadopentetate doses and control group.

Drug dose (*λ*)	O.D (mean ± SD)
Control	0.225 ± 0.08
Gadopentetate 25	0.057 ± 0.003
Gadopentetate 50	0.057 ± 0.008
Gadopentetate 100	0.049 ± 0.003

**Table 6:** The O.D reported by ELASA Reader in 3 nanosized gadopentetate doses and control group.

Drug dose (*λ*)	O.D (mean ± SD)
Control	0.225 ± 0.08
Nanosized gadopentetate 25	0.137 ± 0.010
Nanosized gadopentetate 50	0.139 ± 0.012
Nanosized gadopentetate 100	0.175 ± 0.013

**Table 7:** The O.D reported by ELASA Reader in 3 standard gadopentetate doses, 3 nanosized gadopentetate, and control group.

Drug dose (*λ*)	O.D (mean ± SD)
Control	0.225 ± 0.08
Gadopentetate 25	0.057 ± 0.003
Nanosized gadopentetate 25	0.137 ± 0.010
gadopentetate 50	0.057 ± 0.008
Nanosized gadopentetate 50	0.139 ± 0.012
Gadopentetate 100	0.049 ± 0.003
Nanosized gadopentetate 100	0.175 ± 0.013

**Table 8:** The signals obtained via Dicomworks software and *T*_1_ value calculated by MATLAB software.

TR	1	0.75	0.5	0.25	0.1	Water
20	344	222	233	145	109	17
50	910	645	680	420	279	33
100	1451	1200	1055	730	521	63
200	1717	1572	1475	1230	822	124
400	1781	1685	1642	1651	1497	218
1000	1769	1801	1786	1943	1852	598
2000	1762	1791	1769	1793	2188	1073
3000	1733	1796	1755	1934	2198	1413
1/*T*_1_	0.02734	0.02511	0.02405	0.01416	0.008814	0.001135
*T*_1_ (msec)	36.57	39.82	41.58	70.62	113.45	881.05
*R* ^2^	0.99	0.99	0.987	0.986	0.964	0.984

**Table 9:** The signals obtained via Dicomworks software and *T*_2_ value calculated by Microsoft Excel software.

TE	1	0.75	0.5	0.25	0.1	Water
26	1881	2018	2205	2765	3308	2014
39	1190	1783	1778	2535	3159	2010
52	723	1500	1421	2423	2690	1997
66	531	1233	1158	2302	2681	1979
79	374	1026	922	2070	2656	1954
92	252	847	816	1907	2562	1954
105	152	804	628	1751	2449	1951
118	126	651	581	1572	2245	1935
132	84	506	513	1281	2103	1923
145	70	381	376	1257	1958	1913
1/*T*_2_	0.028	0.014	0.014	0.007	0.004	0.003
*T*_2_ (msec)	35.71	71.42	71.42	142.85	250	333
*R* ^2^	0.99	0.98	0.99	0.99	0.95	0.97

## References

[1] Amanlou M, Siadat SD, Ebrahimi SES (2011). Gd^3+^-DTPA-DG: novel nanosized dual anticancer and molecular imaging agent. *International Journal of Nanomedicine*.

[2] Jemal A, Bray F, Center MM, Ferlay J, Ward E, Forman D (2011). Global cancer statistics. *CA: A Cancer Journal for Clinicians*.

[3] Jemal A, Siegel R, Xu J, Ward E (2010). Cancer statistics, 2010. *CA: A Cancer Journal for Clinicians*.

[4] Fass L (2008). Imaging and cancer: a review. *Molecular Oncology*.

[5] Weishaupt D, Köchli VD, Marincek B (2003). Spin and the nuclear magnetic resonance phenomenon. *How Dose MRI Work? An Introduction to the Physics and Function of the Magnetic Resonance Imaging*.

[6] Reddy JM, Prasad V (2005). MRI contrast agent. *Step by Step MRI*.

[7] Earnshaw A, Greenwood N (1984). The lanthanide elements. *Chemistry of Elements*.

[8] Goldstein HA, Kashanian FK, Blumetti RF, Holyoak WL, Hugo FP, Blumenfield DM (1990). Safety assessment of gadopentetate dimeglumine in U.S. clinical trials. *Radiology*.

[9] Freitas RA (2005). What is nanomedicine?. *Nanomedicine: Nanotechnology, Biology, and Medicine*.

[10] Klajnert B, Bryszewska M (2001). Dendrimers: properties and applications. *Acta Biochimica Polonica*.

[11] Antoni P, Hed Y, Nordberg A (2009). Bifunctional dendrimers: from robust synthesis and accelerated one-pot postfunctionalization strategy to potential applications. *Angewandte Chemie*.

[12] Jain K, Kesharwani P, Gupta U, Jain NK (2010). Dendrimer toxicity: let’s meet the challenge. *International Journal of Pharmaceutics*.

[13] Namazi H, Adeli M (2003). Novel linear-globular thermoreversible hydrogel ABA type copolymers from dendritic citric acid as the A blocks and poly(ethyleneglycol) as the B block. *European Polymer Journal*.

[14] Steger-Hartmann T, Hofmeister R, Ernst R, Pietsch H, Sieber MA, Walter J (2010). A review of preclinical safety data for magnevist (gadopentetate dimeglumine) in the context of nephrogenic systemic fibrosis. *Investigative Radiology*.

[15] Wong K, Ananta JS, lin S, Wilson LJ (2008). In vitro relaxivities studies of gadolinium carbon nanotubes at 3T. *The International Society for Magnetic Resonance in Medicine*.

[16] D’Emanuele A, Attwood D (2005). Dendrimer-drug interactions. *Advanced Drug Delivery Reviews*.

